# BALs are prognostic biomarkers and correlate with malignant behaviors in breast cancer

**DOI:** 10.1186/s12885-025-14576-0

**Published:** 2025-07-24

**Authors:** Xuehao Zhou, Yu Wang, Qingling Xu, Xiang Ao, Mengmeng Chen, Bingqiang Zhang, Ying Liu

**Affiliations:** 1 Institute for Translational Medicine, The Affiliated Hospital of Qingdao University, Qingdao Medical College, Qingdao University, Qingdao, Shandong 266071 China; 2 Department of Urology, Shanghai General Hospital, Shanghai Jiao Tong University School of Medicine, Shanghai, 200080 China; 3 School of Basic Medicine, Qingdao University, Qingdao, Shandong 266071 China; 4 Qingdao Restore Medical Laboratory Co., Ltd., Qingdao, Shandong 266111 China; 5 Key Laboratory of Cancer and Immune Cells of Qingdao, Qingdao, Shandong 266111 China

**Keywords:** Breast cancer, B-aggressive lymphoma protein, Biomarker, Prognosis

## Abstract

**Background:**

The B-aggressive lymphoma (BAL) proteins, including BAL1, BAL2, and BAL3, constitute a conserved protein family characterized by their N-terminal macro domains and putative C-terminal poly (ADP-ribose) polymerase (PARP) active site. Dysregulation of BALs has been closely associated with the progression of various cancers. However, there is limited understanding of their precise expression profile, prognostic significance, and role in breast cancer (BC).

**Methods:**

The expression patterns of BALs were evaluated utilizing multiple databases, including Ualcan, Gene Set Cancer Analysis (GSCA), Search Tool for the Retrieval of Interacting Genes/Proteins (STRING), and Gene Expression Profiling Interactive Analysis (GEPIA). The prognostic significance of BALs was assessed via Kaplan-Meier plotter analysis. Furthermore, the potential mechanisms underlying the contribution of BC progression were predicted through GO and KEGG pathway enrichment analysis. Additionally, the effect of BALs on the malignant behaviors of BC cells was determined using CCK-8 assay, Transwell assay, and TUNEL assay.

**Results:**

The data revealed that the expression levels of both BAL1 and BAL2 were upregulated in BC, whereas no significant change was observed for BAL3. Survival analysis demonstrated a strong association between the overexpression of both BAL1 and BAL2 and favorable prognosis in patients with various subtypes of BC, including estrogen receptor (ER)-positive, ER-negative, Basal, luminal B, HER2-, and HER2 + subtypes. Additionally, the knockdown of BAL1 and BAL2 inhibited the proliferation and migration of BC cells while facilitating apoptosis.

**Conclusions:**

These findings suggest that both BAL1 and BAL2 hold great potential as significant prognostic biomarkers and therapeutic targets for patients with BC.

**Supplementary Information:**

The online version contains supplementary material available at 10.1186/s12885-025-14576-0.

## Introduction

Breast cancer (BC) remains the most prevalent and fatal malignancy among women, accounting for 2,308,897 new cases and 665,684 deaths globally in 2022 [1]. Over an extended period, the incidence and mortality rates of BC have exhibited a significant upward trend, posing a major public health challenge, particularly in developing countries [2]. As a heterogeneous disease, BC encompasses various subtypes characterized by distinct expression patterns of estrogen receptor (ER), progesterone receptor (PR), and human epidermal growth factor receptor 2 (HER2), such as Luminal A, Luminal B, Basal-like, and HER2-positive [3]. Currently, a significant proportion of cases in less developed countries are diagnosed at an advanced stage, leading to a substantial impact on their life expectancy. This difference mainly results from limitations in early detection, prognostic evaluation, and advanced treatment strategies [4]. Multiple biomarkers including tissue markers (e.g., ER, HER-2, Cathepsin D, and p53), genetic markers (e.g., breast cancer gene 1), serum markers (e.g., carcinoembryonic antigen and alpha fetoprotein), as well as certain non-coding RNA markers have been identified to facilitate early diagnosis and prognosis prediction for cancer [5–7]. However, the insufficient specificity and sensitivity of these biomarkers limit their further clinical application. Consequently, there is an urgent need for more reliable biomarkers with enhanced specificity and sensitivity to facilitate early detection and prognosis prediction for BC patients.

The B-aggressive lymphoma (BAL) proteins constitute a conserved protein family characterized by N-terminal macro domains and putative C-terminal poly (ADP-ribose) polymerase (PARP) active sites. In mammals, the BAL family comprises three members: BAL1, BAL2 and BAL3 [8]. BAL1, also known as PARP9, was initially identified in the diffuse large B cell lymphoma due to its capacity to enhance the migration of malignant B cells [9]. BAL2 and BAL3, also known as PARP14 and PARP15, were discovered through sequence alignments of cDNA and protein sequences with human EST, nucleotide, and protein databases related to BAL1 [8]. The PARP domain plays a crucial role in various cellular processes by catalyzing the transfer of ADP-ribose to substrate proteins [10]. However, due to the absence of several key residues, BAL1 lacks the same PARP catalytic activity in both BAL2 and BAL3 [8]. Furthermore, the macro domain primarily regulates transcriptional activities and oncogenesis [11].

Aberrant expression of BALs has been observed in various malignancies, and their dysregulation plays a crucial role in the initiation and progression of cancer [12–14]. For instance, Ma et al. revealed that the circPRKCI/miR-186-5p axis regulates BAL1, thereby promoting the progression and radioresistance of esophageal cancer (EC) [15]. Moore et al. demonstrated that BAL1 and BAL2 are upregulated in pancreatic cancer cells, where they increase NAD(H) consumption via the type I interferon signaling pathway, thereby enhancing the sensitivity of pancreatic cancer cells to nicotinamide phosphoribosyltransferase inhibition [16]. Additionally, Deng et al. highlighted the potential prognostic value of BAL3 in lung adenocarcinoma [17]. However, comprehensive understanding regarding specific expression patterns and exact roles of different members within the BAL family across various subtypes of BC remains unclear. In this study, we utilized online tools to analyze BAL proteins and elucidate their expression levels, pathological characteristics, functional annotations, and prognostic significance in BC. Additionally, we demonstrated that both BAL1 and BAL2 exert inhibitory effects on malignant behaviors of BC cells. Our findings strongly support the potential utility of BAL1 and BAL2 as promising biomarkers and therapeutic targets for BC.

## Materials and methods

### Gene expression profiling interactive analysis database analysis

The Gene Expression Profiling Interactive Analysis database (GEPIA) [18] (http://gepia.cancer-pku.cn/) is an online database containing The Cancer Genome Atlas (TCGA) and GTEx data, enabling researchers to conduct various investigations such as differential expression analysis, prognosis analysis, and correlation analysis. We used GEPIA to perform the expression profile and correlation analysis of BALs.

### Gene set cancer analysis database analysis

The Gene Set Cancer Analysis database (GSCA) (http://bioinfo.life.hust.edu.cn/GSCA/#/) is an openly accessible online database that provides access to gene expression analysis, immune analysis, mutation analysis, and drug sensitivity analysis. In this study, we use GSCA database to determine the different expression of BAL family between cancer samples and normal samples.

### The encyclopedia of RNA interactomes database analysis

The Encyclopedia of RNA Interactomes (ENCORI) [19] (https://rnasysu.com/encori/) is a comprehensive database that provides the analysis of RNA-RNA and protein-RNA interactions, as well as pan-cancer analysis consisting of 32 different cancer types derived from TCGA data. We utilized ENCORI to detect the differential expression levels of BALs between normal tissue and BC tissue.

### University of Alabama cancer database analysis

The University of Alabama Cancer (Ualcan) database [20] (http://ualcan.path.uab.edu/analysis.html), established based on the TCGA project, offers a platform for researchers to analyze the expression patterns of approximately 20,500 protein-coding genes in 33 types of malignancies. By utilizing this valuable resource, we investigated the differential expression profiles of BAL family members between primary tumor tissues and corresponding normal tissues. Additionally, we explored the expression characteristics of BAL1 and BAL2 in patients with diverse features including age groups, subtypes, cancer stages, and genders.

### Survival analysis

The Kaplan-Meier plotter [3] (https://kmplot.com/analysis/) is an openly accessible online database that enables the analysis of the impact of specific genes on patients’ survival across diverse tumor types, including various cancer subtypes. In this study, we utilized this database to investigate the prognostic significance of BAL1 and BAL2 in BC patients by assessing their relapse-free survival (RFS). The method for dividing patients into high and low expression groups was determined based on the median value. The *p*-values were adjusted using the log-rank test.

### Search tool for the retrieval of interacting genes/proteins database analysis

The Search Tool for the Retrieval of Interacting Genes/Proteins (STRING) database [21] (https://cn.string-db.org/) is a comprehensive resource developed for integrating both known and predicted protein-protein interactions, encompassing physical interactions and functional associations. In this study, we utilized the STRING database to construct a network comprising BAL1, BAL2, and their closely associated genes.

### SRplot platform analysis

SRplot (http://www.bioinformatics.com.cn/en) is an online platform utilized for data visualization and graphing. To predict the potential functions of BALs and their most correlated proteins, we performed Gene ontology (GO) and Kyoto Encyclopedia of Genes and Genomes (KEGG) enrichment analysis using SRplot.

### Database for annotation, visualization and integrated discovery database analysis

The Database for Annotation, Visualization and Integrated Discovery (DAVID) is an online bioinformatic database that serves as a valuable tool for investigating the function of differential expressed genes and conducting enrichment analysis. In this study, we utilized the DAVID database to analyze BAL1, BAL2, and their co-expressed genes to visualize their representation on KEGG pathway maps.

### Cell culture and real-time quantitative polymerase chain reaction analysis

The human BC cell line MCF-7 and MDA-MB-231 was obtained from Cellverse Bioscience Technology (Shanghai, China), as previously utilized in our prior investigation [22]. Cells were cultured in Dulbecco’s modified Eagle’s medium (DMEM, Invitrogen, USA) supplemented with 10% fetal bovine serum (FBS) (Hyclone, USA) and penicillin-streptomycin (100 U/ml penicillin and 0.1 mg/ml streptomycin) at 37 °C under a 5% CO_2_ atmosphere. SiRNAs (NC, BAL1, and BAL2) were obtained from Gene Pharma (Shanghai, China).

MCF-7 cells were transfected with appropriate siRNAs. Total RNA was extracted from the cells using an RNA isolater Total RNA Extraction Reagent (Vazyme, Nanjing, China), and then subjected to reverse transcription using a HiScript III Q RT SuperMix for qPCR (+ gDNA wiper) kit (Vazyme, Nanjing, China). The mRNA levels of BAL1, BAL2, and GADPH (as an internal control) were quantitated by real-time quantitative polymerase chain reaction (RT-qPCR) using a SYBR Green Realtime PCR Master Mix (Vazyme, Nanjing, China). The following primer sequences were used: BAL1: 5’-CCTTTGGCGCTCGTTAGGA-3’ (forward) and 5’-ACCTGCAAACCACATTTTTCAA-3’ (reverse); BAL2: 5’-GCCTGCAGATGCTGTTGGT-3’ (forward) and 5’-GCCACCCCTTGTCCAACTG-3’ (reverse); GAPDH: 5’-TGGAGTCTACTGGCGTCTT-3’ (forward) and 5’- TGTCATATTTCTCGTGGTTCA − 3’ (reverse) (synthesized by Invitrogen).

### Western blot analysis

The protein supernatants were extracted from cells transfected with appropriate siRNAs, and subsequently subjected to SDS-PAGE. After electrophoresis, the protein bands were transferred onto PVDF membrane and incubated with primary antibodies. Following incubation with corresponding secondary antibodies, BAL1 and BAL2 proteins were detected using the Omin-ECL Ultra-sensitive Chemiluminescence Detection kit (EpiZyme, Shanghai, China). Image J 6.0 software was employed for signal intensity quantification. Primary antibodies used included anti-BAL1 (17535-1-AP, proteintech, China) and anti-BAL2 (30127-1-AP, proteintech, China), and anti-β-actin (AC026, Abclonal, China). Secondary antibody utilized was HRP-conjugated Goat anti-Rabbit IgG (H + L) antibody (AS014, Abclonal, China).

### Cell proliferation analysis

The proliferation of MCF-7 cells was evaluated using the Cell Counting Kit-8 (CCK-8) assay. MCF-7 cells were seeded at a density of 10^3^−10^4^ cells per well in a 96-well plate. After transfection with corresponding siRNAs, the cells were cultured for 0, 24, 48, 72, or 96 h. Subsequently, each well was supplemented with 10 µL of CCK-8 solution (GK10001, GLPBIO, USA) and further incubated for an additional hour. Optical density (OD) values were then measured at an absorbance of 450 nm using SpectraMax iD3/iD5 multifunctional ELISA reader (Molecular Devices, USA).

### Cell migration analysis

The migration capacity of MCF-7 cells was assessed using Transwell chambers (Corning, NY, USA). The lower chamber was supplemented with DMEM containing 10% FBS as a chemoattractant. Following transfection with specific siRNAs, the cells were seeded at a density of 8 × 10^4^ cells in the upper chamber and incubated for 24 h at 37 °C in the presence of 5% CO_2_ using 200 µL serum-free medium. Subsequently, the cells were fixed with 70% ethanol, stained with 0.1% crystal violet, and observed for invasive cells using an invert microscope (Olympus, Japan).

### Cell apoptosis analysis

The apoptosis of MCF-7 cells was evaluated using a TUNEL Detection Kit (40406ES20, Yisheng China). Apoptotic cells were identified by TUNEL-positive signals, while the total cell count was determined by blue DAPI-stained nuclei. Signal intensity quantification was performed using Image J 6.0 software. The apoptotic rate of MC-7 cells was calculated by dividing the number of positive cells per field of view by the total cell count and multiplying it by 100%.

### Statistical analysis

The differential expression levels of BALs between BC samples and normal samples were assessed using Student’s t-tests, while the expression of BALs in patients with different clinical characteristics was also examined. Survival curves for different BC subtypes, based on varying expression levels of either BAL1 or BAL2, were generated through log-rank tests and hazard ratios (HRs) from the Kaplan-Meier plotter analysis. A significance level of *p* < 0.05 was considered statistically significant.

## Results

### BAL1 and BAL2 are significantly upregulated in patients with BC

**Fig. 1:**
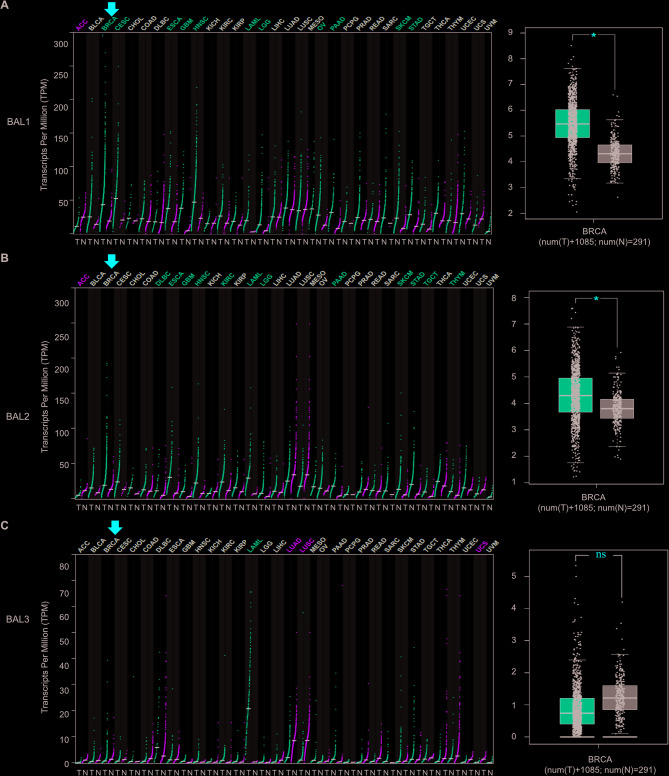
Pan-cancer analysis of BAL family expression across 33 cancer types. The expression levels of BAL1 (**A**), BAL2 (**B**), and BLA3 (**C**) in BC tissue samples and normal tissue samples were obtained from the GEPIA database. The red arrows represent BC tissues, while the grey columns represent normal tissues. A total of 1085 BC tissue samples and 291 normal tissue samples were included to investigate BAL expression levels. The y-axis indicates the logarithm of gene expression in the samples. *p < 0.05 indicates a significant difference between normal tissue samples and BC tissue samples. BAL, B-aggressive lymphoma; GEPIA, gene expression profiling interactive analysis

**Fig. 2:**
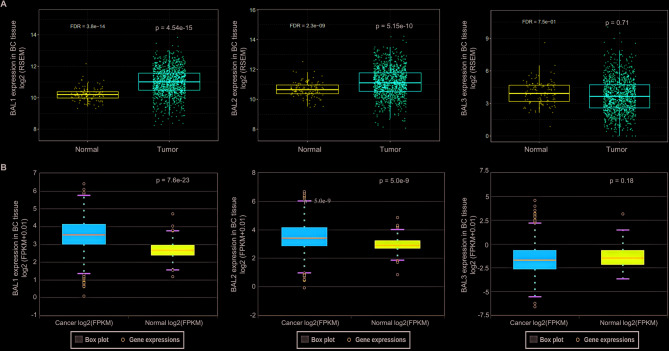
Expression profiles of BAL family in BC based on GEPIA database (**A**) and ENCORI (**B**) database. The GSCA database comprised 1104 BC tissue samples and 114 normal tissue samples, whereas the ENCORI database included data from 1104 BC tissue samples and 113 normal tissue samples. BAL, B-aggressive lymphoma; BC, breast cancer; GEPIA, gene expression profiling interactive analysis; ENCORI, encyclopedia of RNA interactomes

To date, three members of the BAL family (BAL1, BAL2, and BAL3) have been identified as playing a role in the progression various cancers. Although overexpression of BAL1 has been reported in BC, the precise expression patterns and functional roles of other members within the BAL family in BC remain largely unknown. To investigate their potential involvement in BC progression, we conducted a pan-cancer analysis using the GEPIA database to assess their expression profiles. As shown in Fig. 1, the mRNA levels of both BAL1 and BAL2 were significantly upregulated in BC tissues compared with adjacent normal tissues, whereas no significant difference was observed in BAL3 expression between BC tissues and adjacent normal tissues. Furthermore, to explore individual member expression patterns of BAL family members in BC, we performed an analysis using GSCA database with 114 paired samples. No significant difference was observed for BAL3 expression (*p* = 0.71) between BC tissues and adjacent normal tissues, whereas both BAL1 and BAL2 exhibited highly significant differences with p values of 4.54 × 10^−15^ and 1.03 × 10^−29^ respectively, indicating strong statistical significance (Fig. 2A). The concordance of BAL expression profiles among BC patients was validated using the ENCORI database (Fig. 2B), confirming significant upregulation of both BAL1 and BAL2 in BC.

### Analysis of the expression patterns of BAL1 and BAL2 in BC patients with distinct clinical characteristics

**Fig. 3:**
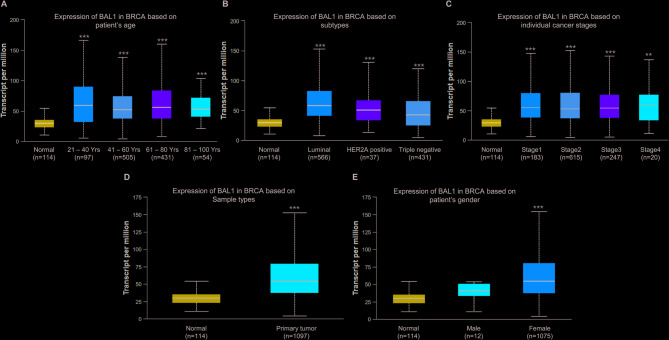
Analysis of BAL1 expression patterns in BC patients with distinct pathologic characteristics. Ualcan analysis revealed the transcriptional levels of BAL1 in BC patients with different age (**A**), cancer subtypes (**B**), cancer stages (**C**), sample types (**D**) and gender (**E**). Subtype composition: Normal samples: 114, Luminal subtype: 566, HER2 + subtype: 37, Triple-negative subtype: 116. The statistical significance between each group with normal sample was represented with asterisk (*p < 0.05, **p < 0.01, ***p < 0.001). BAL1, B-aggressive lymphoma 1; BC, breast cancer; Ualcan, University of Alabama cancer

**Fig. 4:**
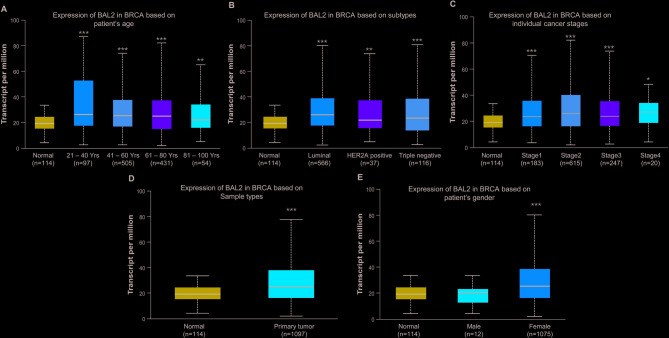
Analysis of BAL2 expression patterns in BC patients with distinct pathologic characteristics. Ualcan analysis revealed the transcriptional levels of BAL2 in BC patients with different age (**A**), cancer subtypes (**B**), cancer stages (**C**), sample types (**D**) and gender (**E**). Subtype composition: Normal samples: 114, Luminal subtype: 566, HER2 + subtype: 37, Triple-negative subtype: 116. The statistical significance between each group with normal sample was represented with asterisk (*p < 0.05, **p < 0.01, ***p < 0.001). BAL2, B-aggressive lymphoma 2; BC, breast cancer; Ualcan, University of Alabama cancer

Considering the observed overexpression of BAL1 and BAL2 in BC tissues, we hypothesized a potential correlation between their expression patterns and clinical characteristics of BC patients. To investigate this possibility, we performed an analysis using the Ualcan database. As shown in Fig. 3, significant differences were observed in BAL1 expression across various clinical parameters including age, molecular subtypes, stages, sample types, and sex among BC patients. Notably, compared to the normal group, all patient groups with distinct ages exhibited significantly increased BAL1 expression (Fig. 3A). Furthermore, elevated levels of BAL1 were observed across different molecular subtypes of BC, specifically Luminal, HER2-positive, and triple-negative subtypes. Notably, no significant differences in BAL1 expression were observed between the luminal subtype and the HER2-positive subtype or between the HER2-positive subtype and the triple-negative subtype. In contrast, a significant difference in BAL1 expression was identified between the luminal subtype and the triple-negative subtype (*p* = 2.75 × 10^−6^) (Fig. 3B). Consistent trends were also observed when analyzing individual cancer stages and sample types separately (Fig. 3C and D). Strikingly, BAL1 transcriptional level showed exclusive elevation in female patients with BC, but not male patients (Fig. 3E). Similarly, analogous results were observed for the expression pattern of BAL2 in BC patients with corresponding clinical characteristics, except that no significant differences were observed in the expression levels of BAL2 among the distinct subtypes (Fig. 4). Collectively, these findings strongly support the notion that both BAL1 and BAL2 play critical roles in the progression of BC exhibiting diverse clinical characteristics.

### High expression of BAL1 and BAL2 is significantly associated with favorable prognosis of patients with BC

**Fig. 5:**
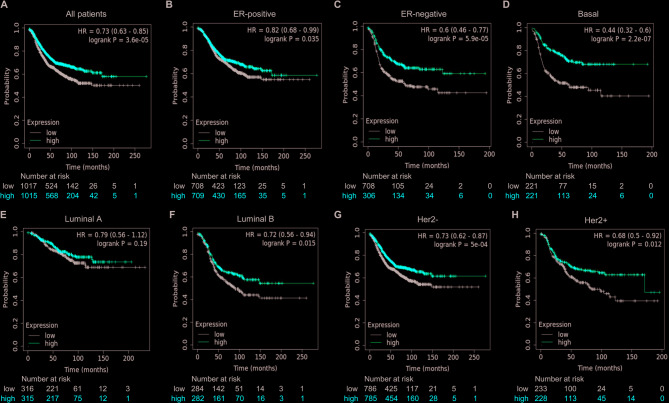
Prognostic significance of BAL1 expression in BC patients with different molecular subtypes. Kaplan-Meier plots were conducted to draw the RFS curves for all patients (*n* = 2032) (**A**), ER-positive (*n* = 1417) (**B**), ER-negative (*n* = 615) (**C**), Basal-like (*n* = 442) (**D**), Luminal A-like (*n* = 631) (**E**), Luminal B-like (*n* = 566) (**F**), HER2-negative (*n* = 1571) (**G**) and HER2-positive (*n* = 461) (**H**) subtypes. BAL1, B-aggressive lymphoma 1; BC, breast cancer; ER, estrogen receptor; HER2, human epidermal growth factor receptor 2

**Fig. 6:**
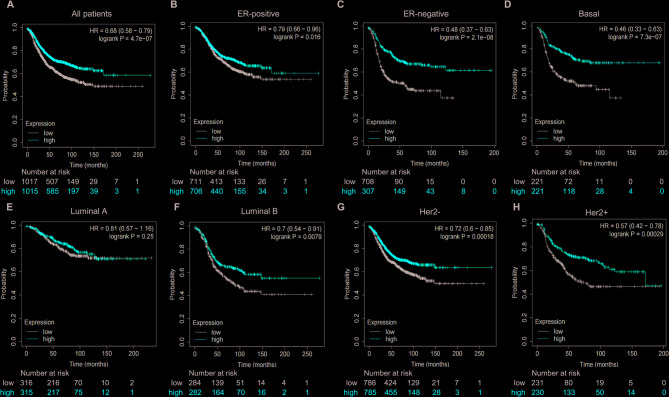
Prognostic significance of BAL2 expression in BC patients with different molecular subtypes. Kaplan-Meier plots were conducted to draw the RFS curves for all patients (*n* = 2032) (**A**), ER-positive (*n* = 1417) (**B**), ER-negative (*n* = 615) (**C**), Basal-like (*n* = 442) (**D**), Luminal A-like (*n* = 631) (**E**), Luminal B-like (*n* = 566) (**F**), HER2-negative (*n* = 1571) (**G**) and HER2-positive (*n* = 461) (**H**) subtypes. BAL2, B-aggressive lymphoma 2; BC, breast cancer; ER, estrogen receptor; HER2, human epidermal growth factor receptor 2

We further assessed the prognostic significance of BAL1 and BAL2 in BC and generated corresponding survival curves using Kaplan Meier plotter analysis. As shown in Fig. 5A, high expression of BAL1 was significantly associated with longer RFS for all BC patients (HR = 0.73, *p* = 3.6 × 10^−5^). Moreover, subtype-specific analysis of BC revealed that high expression of BAL1 was significantly correlated with prolonged RFS in ERpositive (HR = 0.82, *p* = 0.035), ERnegative (HR = 0.6, *p* = 5.9 × 10^−5^), Basal (HR = 0.44, *p* = 2.2 × 10^−7^), luminal B (HR = 0.72, *P* = 0.015), HER2- (HR = 0.73, *p* = 5 × 10^−4^), and HER2+ (HR = 0.68, *p* = 0.012) subtypes, but not in the luminal A subtype (HR = 0.79, *p* = 0.19) (Fig. 5B-H). Similarly to the findings for BAL1, high levels of BAL2 were significantly associated with extended RFS across all BC patients (HR = 0.68, *p* = 4.7 × 10^−7^), as well as within ER-positive (HR = 0.79, *p* = 0.016), ER negative (HR = 0.48, *p* = 2.1 × 10^−8^), Basal(HR = 0.46, *p* = 7.3 × 10^−7^), luminal B (HR = 0.7, *p* = 0.0079), HER2- (HR = 0.72, *p* = 0.00018), HER2+ (HR = 0.57, *p* = 0.00029) subtypes, but not within luminal A subtype (HR = 0.81, *p* = 0.25) (Fig. 6A-H). These results suggest that both BAL1 and BAL2 may serve as general biomarkers indicative of a relatively favorable prognosis in BC.

### The predictive function and pathways of BAL1 and BAL2 in BC

**Fig. 7:**
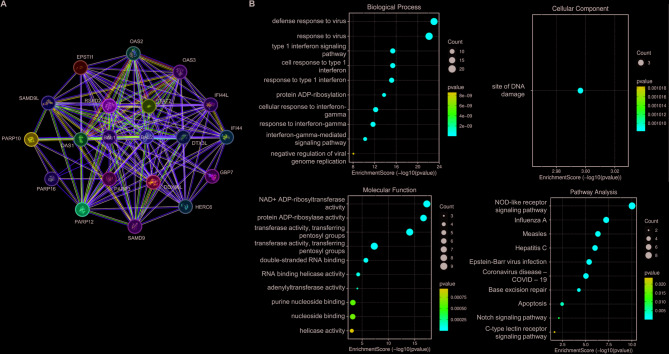
Protein interaction network and functional annotation analysis of BALs in BC. **A** Protein-protein interaction network of BALs and their associated co-expressed genes was conducted by STRING analysis. **B** GO and KEGG pathway enrichment analysis on SRplot platform was performed to predict the potential functions of BALs and their co-expressed proteins, including biological process, cellular component, molecular function, as well as pathway. BAL, B-aggressive lymphoma; BC, breast cancer; STRING, search tool for the retrieval of interacting genes/proteins; GO, gene ontology; KEGG, Kyoto encyclopedia of genes and genomes

Using the STRING database, we constructed a protein interaction network of BAL1 and BAL2 comprising 20 correlated genes, including PARP3, DTX3L, RSAD2, DDX60L, USP18, SAMD9L, and OAS1 (Fig. 7A). Subsequently, GO and KEGG analysis on SRplot platform was employed to predict the potential functions of BAL1, BAL2, and their co-expressed genes in terms of biological processes, cellular components and molecular functions as well as pathways. As shown in Fig. 7B, alterations in BAL1 and BAL2 played crucial roles in regulating various biological processes, including defense response to virus, response to virus, type I interferon signaling pathway, cellular response to type I interferon, response to type I interferon, protein ADP-ribosylation, cellular response to interferon-gamma, response to interferon-gamma, interferon-gamma-mediated signaling pathway, and negative regulation of viral genome replication. Furthermore, the alterations of BAL1 and BAL2 affected the site of DNA damage at a cellular level (Fig. 7C). Additionally, BAL1 and BAL2 were involved in the modulation of NAD + ADP-ribosyltransferase activity, protein ADP-ribosylase activity, transferase activity and transferring pentosyl groups, transferase activity and transferring glycosyl groups, double-stranded RNA binding, RNA helicase activity, adenylyltransferase activity, purine nucleoside binding, nucleoside binding, as well as helicase activity (Fig. 7D).

To gain a more comprehensive understanding of the underlying mechanisms of BALs involved in the progression of BC, we conducted KEGG analysis using the SRplot platform to identify pathways associated with alterations in BAL1 and BAL2 expression, as well as their co-expressed genes (Fig. 7E). Among these pathways, Nucleotide-binding and oligomerization domain (NOD)-like receptor signaling and apoptosis signaling pathways have been extensively implicated in cancer initiation and development [23–25]. These pathway maps along with their associated co-expressed genes were visualized using the DAVID database (Fig. S1 and S2).

### BAL1 and BAL2 facilitated the malignant behaviors of BC cells

**Fig. 8:**
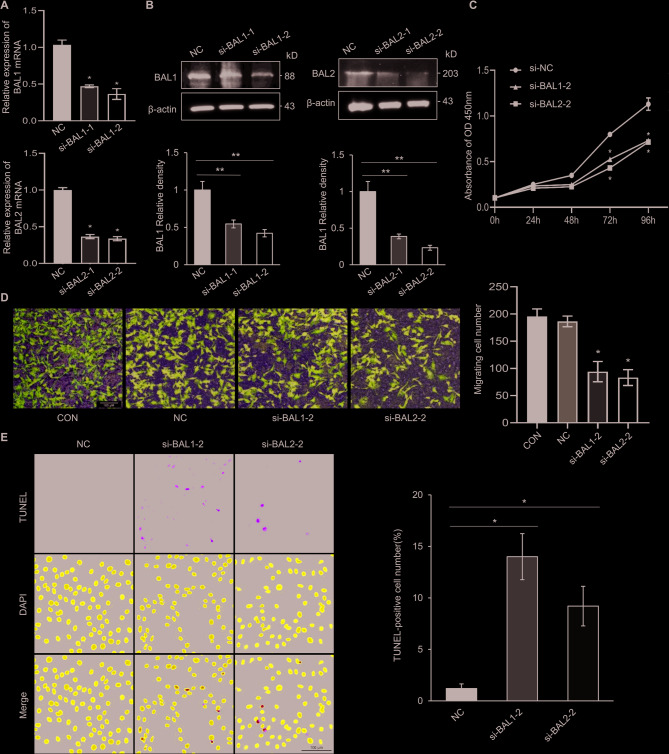
Effect of BAL1 and BAL2 on malignant behaviors of MCF-7 cells. MCF-7 cells were transfected with siNC, siBAL1-1, siBAL1-2, siBAL2-1, or siBAL2-2 and subsequently subjected to RT-qPCR assay (**A**), Western blot assay (**B**), CCK-8 assay (**C**), Transwell assay (**D**), and TUNEL assay (**E**). Each experiment was repeated at least three times. Data are presented as means ± SDs from three independent experiments *p < 0.05. BAL1, B-aggressive lymphoma 1; BAL2, B-aggressive lymphoma 2; BC, breast cancer; RT-qPCR, real-time quantitative polymerase chain reaction; CCK-8, cell counting kit-8

**Fig. 9:**
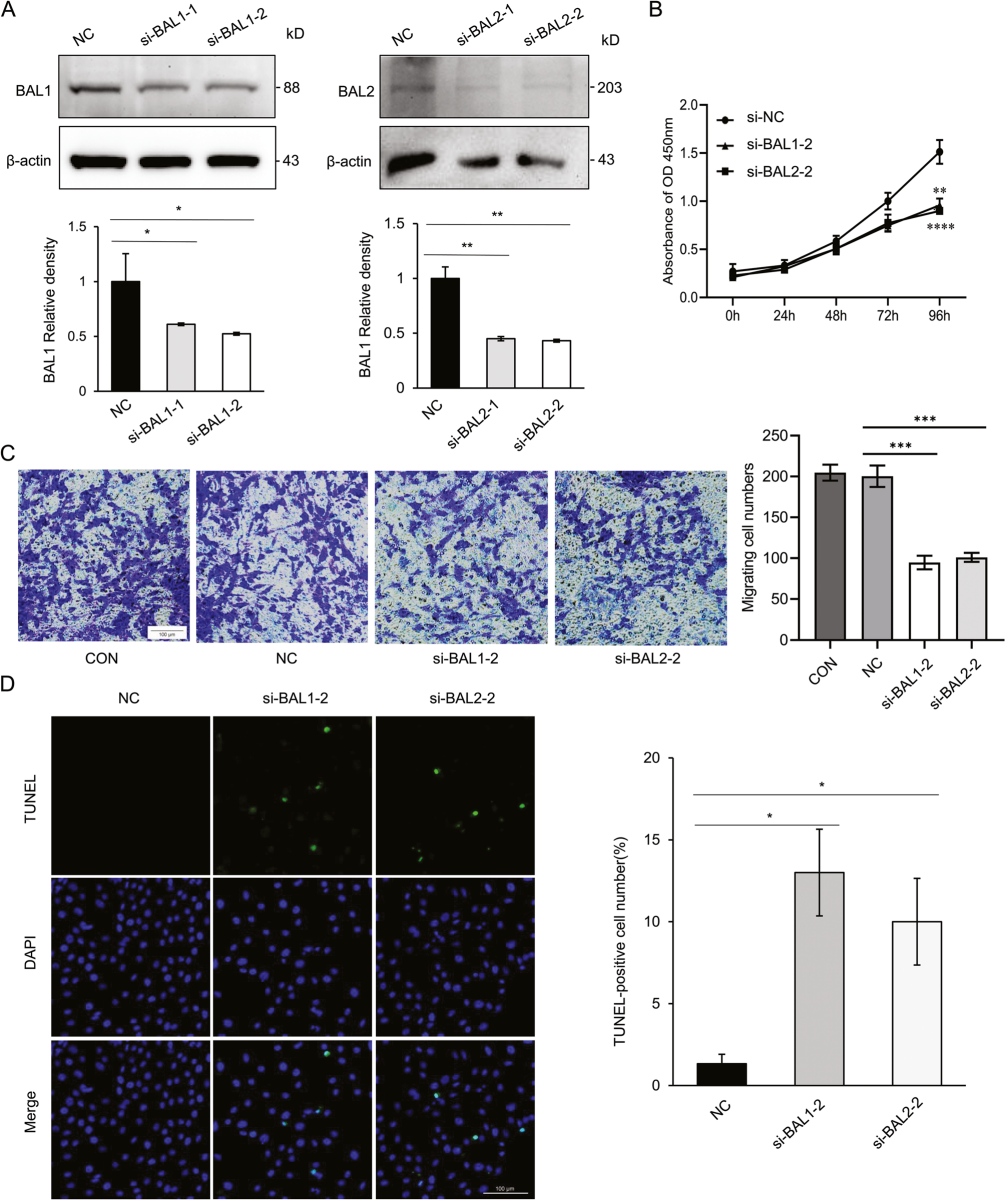
Effect of BAL1 and BAL2 on malignant behaviors of MDA-MB-231 cells. MDA-MB-231 cells were transfected with siNC, siBAL1-1, siBAL1-2, siBAL2-1, or siBAL2-2 and subsequently subjected to Western blot assay (**A**), CCK-8 assay (**B**), Transwell assay (**C**), and TUNEL assay (**D**). Each experiment was repeated at least three times. Data are presented as means ± SDs from three independent experiments **p < 0.01, ***p < 0.001, ****p < 0.0001. BAL1, B-aggressive lymphoma 1; BAL2, B-aggressive lymphoma 2; BC, breast cancer; RT-qPCR, real-time quantitative polymerase chain reaction; CCK-8, cell counting kit-8

The upregulation of BAL1 and BAL2 in BC tissues indicates their involvement in regulating BC progression. To further investigate their functional effects, we performed CCK-8 and Transwell analysis to evaluate the effect of BAL1 and BAL2 on the proliferation and migration of BC cells. Two specific siRNAs targeting BAL1 or BAL2 were synthesized and validated for their knockdown efficiency. MCF-7 cells were divided into different groups and transfected with corresponding siRNAs. qRT-PCR and Western blog analysis demonstrated that siBAL1-2 and siBAL2-2 exhibited superior efficacy in downregulating BAL1 and BAL2 expression in MCF-7 cells (Fig. 8A and B). Consequently, these two siRNAs were used for subsequent experiments. CCK-8 analysis revealed that transfection with either siBAL1-2 or siBAL2-2 significantly inhibited the proliferation of MCF-7 cells (Fig. 8C). Furthermore, Transwell analysis indicated a marked reduction in migratory capacity following knockdown of BAL1 or BAL2in MCF-7 cells (Fig. 8D). Additionally, TUNEL assay confirmed that silencing BAL1 and BAL2 remarkably facilitated apoptosis in MCF-7 cells (Fig. 8E). To validate these findings, we extended our investigation to another BC cell line, MDA-MB-231. Consistent results were obtained, confirming the promoting effects of BAL1 and BAL2 on malignant behaviors in BC cells (Fig. 9). Collectively, these data strongly support the notion that BAL1 and BAL2 may function as critical regulators in driving the malignant progression of BC.

## Discussion

The high global morbidity of BC establishes it as the most frequently diagnosed cancer in women, seriously threatening patients’ lives and overall health [26]. Despite significant advancements in therapeutic strategies, BC remains a formidable barrier to extending female life expectancy. The pathogenesis of BC is highly complicated, involving diverse biological processes and exhibiting an unfavorable prognosis [27]. In clinical practice, it is crucial to assess the prognostic status and therapeutic efficacy of cancer patients. However, patient outcomes remain poor due to the lack of effective assessment approaches [28]. Multiple biomarkers, such as nucleic acid, protein, and extracellular vesicle, have been utilized for prognostic evaluation in cancer treatment, but their limited specificity and sensitivity hinder their clinical utilization [3, 29, 30]. Therefore, it is an urgent need to screen and identify novel prognostic biomarkers that exhibit high specificity and sensitivity in BC treatment. This study comprehensively analyzed expression patterns, pathological characteristics, functional roles, and prognostic significance of BALs in BC using publicly accessible databases with the aim of enhancing prognostic accuracy and optimizing BC treatment strategies.

BAL proteins have been demonstrated to play crucial roles in the progression of various cancers, including cervical cancer, colon cancer (CC), and acute myeloid leukemia (AML). For instance, Tao et al. revealed that BAL1 mediated the oncogenic function of lncSNHG16 in cervical cancer. Overexpression of BAL1 counteracted the effects induced by lncSNHG16 knockdown on the proliferation and invasion of cervical cancer cells [31]. Mashimo et al. discovered that inhibition of BAL2 in CC cells resulted in reduced mRNA expression levels of E type prostanoid 4 receptors, which are essential regulators involved in the progression and malignant transformation of CC [32]. Furthermore, Zhu et al. showed that BAL2 facilitated HIF-1α expression by activating NF-κB signaling pathway, leading to AML cell proliferation promotion and glycolysis enhancement while suppressing apoptosis [33]. It has been reported that BAL1 was upregulated in BC tissues, and its knockdown suppressed BC cell migration [34]. However, the specific expression profile of other members within the BAL family remains unclear for BC. In this study, data from GEPIA and GSCA databases revealed a significant upregulation of BAL1 and BAL2 mRNA expression levels in BC tissues, while no significant change was observed for BAL3 (Figs. 1 and 2). By stratifying patients based on clinicopathological characteristics using Ualcan analysis, we further investigated the expression patterns of BAL1 and BAL2 in BC patients. Our results demonstrated a significant upregulation of both BAL1 and BAL2 mRNA expressions across different age groups, molecular subtypes, stages, sample types, and sexes (Figs. 3 and 4). These findings strongly support the notion that both BAL1 and BAL2 are overexpressed in BC tissues and their expression is closely associated with disease progression, highlighting their unique role in BC. Notably, in our subtype analysis, we found that BAL1 expression was higher in the luminal subtype than in the triple-negative BC subtype. This suggests that the function of BAL1 may be linked to the ER or PR pathway. In contrast, no significant differences were observed in BAL2 expression among the three subtypes, indicating that BAL2 may have a distinct mechanism in BC compared with BAL1 and that its function is independent of the ER or PR pathway. However, the precise mechanisms underlying the involvement of BAL1 and BAL2 in BC remain unclear.

BAL proteins have demonstrated significant prognostic significance in various cancer types, including glioma, glioblastoma and AML. For instance, Xu et al. identified BAL1 as a promising prognostic biomarker and immunotherapeutic target for glioma [35]. Zhang et al. revealed that BAL2 was associated with poor prognosis and could serve as an independent prognostic biomarker for glioblastoma [36]. Lee et al. demonstrated the close association between two BAL3 polymorphisms and improved overall survival in AML patients, suggesting the potential utility of BAL3 polymorphism as a prognostic biomarker in AML treatment [14]. To confirm the prognostic value of BAL1 and BAL2 in BC patients, we performed Kaplan–Meier plotter analysis to access the RFS curves across different molecular subtypes of BC (Figs. 5 and 6). High expression levels of both BAL1 and BAL2 were significantly associated with favorable prognosis in BC patients. Specifically, within ER-positive, ER-negative, Basal-like, luminal B, HER2- and HER2 + subtypes of BC, high expression levels of both BAL1 and BAL2 were correlated with better prognosis outcomes. These findings suggest that both BAL1 and BAL2 may hold great potential for prognosis assessment and treatment strategies specifically tailored to these BC subtypes. However, their prognostic value in luminal A subtype remains uncertain.

To further investigate the role and underlying mechanism of BAL1 and BAL2 in BC, we constructed a protein-protein interaction network using the STRING database to identify potential interacting proteins with BAL1 and BAL2 (Fig. 7). Our results revealed strong associations between BAL1 and BAL2 with DTX3L, RSAD2, OAS1, PARP3, and DDX60L. Notably, most of these proteins have been implicated in critical processes involved in BC. For instance, DTX3L regulates both LIPG signaling and the functionality of all-trans retinoic acid in the progression of BC [37, 38]. RSAD2 served as an independent risk factor capable of predicting tumor stage, grade, and lymph node metastases in BC [39]. Furthermore, decreased levels of STAT2 hampered the anti-tumor immune response in luminal BC by affecting the activation of type I IFN signaling upon inhibition of EZH2 [40]. Additionally, OAS1 promoted BC cell proliferation and resistance to tamoxifen through a non-coding RNA-mediated mechanism [41, 42]. These findings suggest that the involvement of BAL1 and BAL2 in BC progression may be attributed to their interaction with these proteins. Moreover, our data from GO and KEGG analysis identified multiple pathways associated with altered function of BAL1 and BAL2 in BC, including base excision repair, NOD-like receptor signaling pathway, and apoptosis (Figs. 7B, 8 and 9). Peng et al. demonstrated that the NOD-like receptor signaling pathway played a crucial role in mediating the ability of USP21 to facilitate cell proliferation, migration, and invasion in triple negative BC [43]. Sethy et al. revealed that BMN-673, a PARP inhibitor, effectively inhibited base excision repair by modulating pol-β within chromatin inducing apoptosis in BC cells [44]. Furthermore, BAL1 was found to suppress apoptosis in diffuse large B-cell lymphoma through IFNγ-STAT1-IRF1-p53 axis [45]. We subsequently visualized the positions of BAL1 and BAL2-related genes within key pathways using the DAVID database. As shown in Fig. 8, IRF9, a gene associated with the BAL family, can interact with STAT1 to activate interferon-stimulated genes, thereby contributing to the biological effects of interferons [46]. Previous studies have demonstrated that this signaling is involved in BC progression and drug resistance [47, 48]. Besides, the OAS protein family has been reported to play a critical role in antiviral responses, interferon signaling, and inflammatory tumor microenvironment. In BC, these proteins are significantly upregulated, leading to increased proliferation and unfavorable prognosis [49, 50]. BAL1 and BAL2 may modulate the activity of the NOD-like receptor pathway through their associated genes, thereby promoting the malignant behaviors of BC cells. In the apoptosis pathway, CASPs belong to a protein family involved in programmed cell death, inflammatory responses, and immune processes. Specifically, CASP2, CASP8, CASP9, and CASP10 serve as initiator caspases in cell death, while CASP3, CASP6, and CASP7 function as downstream effectors [51]. These caspases have also been shown to regulate proliferation and cell death in BC [52, 53]. These findings provide novel directions for elucidating the mechanisms underlying the role played by BALs in BC progression.

To address the biases inherent in bioinformatics analysis, we conducted in vitro experiments to validate the impact of BAL1 and BAL2 on malignant behaviors of BC cells. Our findings unequivocally demonstrate that knockdown of BAL1 and BAL2 significantly suppresses proliferation and migration, while facilitating apoptosis in BC cells compared to control cells (Figs. 8 and 9). These findings strongly support a pivotal role for BAL1 and BAL2 in driving BC progression. However, certain limitations exist within in this study. For instance, our investigation primarily relied on bioinformatics analysis to elucidate the underlying mechanisms of BALs, these mechanisms were not experimentally validated at the molecular level. Furthermore, we did not explore the potential involvement of BALs across different subtypes of BC. Additionally, the knockdown of BAL1 and BAL2 resulted in reduced BC cell proliferation and migration, suggesting that BAL1 and BAL2 may promote the malignant behaviors of BC cells. However, their high expression was closely associated with a favorable prognosis in BC patients. The reasons for this phenomenon may be as follows: (1) BAL1 and BAL2 may exhibit dual functionality due to variations in the tumor microenvironment or differences across stages of BC progression. (2) BAL1 and BAL2 may possess immune-modulatory properties. While their high expression promotes BC cell proliferation and migration, they may concurrently enhance anti-tumor immune responses, thereby contributing to an improved clinical outcome. (3) Sampling heterogeneity may account for this discrepancy. Our prognostic analysis was based on whole-tissue sample data from public databases, whereas the migratory effects were observed in vitro using cell lines, potentially reflecting localized expression patterns.

In conclusion, we conducted a comprehensive investigation into the expression pattern, clinicopathological characteristics, functional roles, and prognostic significance of BALs in BC. We observed upregulation of both BAL1 and BAL2 in BC tissues compared to normal tissues, while no significant difference was observed for BAL3. Importantly, high expression levels of both BAL1 and BAL2 were strongly associated with favorable prognosis across all patient groups as well as specific subtypes including ER-positive, ER-negative, Basal-like, luminal B-like, HER2- and HER2 + BC. These findings strongly suggest that BAL1 and BAL2 possess potential as prognostic biomarkers for BC. Furthermore, both BAL1 and BAL2 significantly promoted the malignant behaviors of BC cells. Further investigations are warranted to elucidate the biological effects of BAL1 and BAL2 in specific contexts through gene knockout or overexpression experiments during BC progression. Additionally, integrating immunohistochemical analysis or single-cell sequencing techniques should be performed to comprehensively investigate their spatial heterogeneity and functional diversity.

## Supplementary Information

Supplementary Material 1.

Supplementary Material 2.

## Data Availability

The data analyzed in this study can be downloaded from the TCGA (https://portal.gdc.cancer.gov/).

## Abbreviations

BAL: B-aggressive lymphoma
ER: Estrogen receptor
BC: Breast cancer
HER2: Human epidermal growth factor receptor 2
PARP: Poly (ADP-ribose) polymerase
EC: Esophageal cancer
GEPIA: Gene Expression Profiling Interactive Analysis database
TCGA: The Cancer Genome Atlas
GSCA: Gene Set Cancer Analysis database
ENCORI: Encyclopedia of RNA Interactomes
Ualcan: University of Alabama Cancer
STRING: Search Tool for the Retrieval of Interacting Genes/Proteins
GO: Gene ontology
KEGG: Kyoto Encyclopedia of Genes and Genomes
DAVID: Database for Annotation, Visualization and Integrated Discovery
CCK-8: Cell Counting Kit-8
OD: Optical density
HR: Hazard ratio
NOD: Nucleotide-binding and oligomerization domain
AML: Acute myeloid leukemia
