# Supplementary material for: BALs are prognostic biomarkers and correlate with malignant behaviors in breast cancer
Source: BMC Cancer. 2025 Jul 24;25:1205. doi: 10.1186/s12885-025-14576-0 (PMC12288338; doi:10.1186/s12885-025-14576-0)
Supplement: Supplementary file 1 — Supplementary Material 1. [file 12885_2025_14576_MOESM1_ESM.docx]

Supplementary Materials

**BALs are prognostic biomarkers and correlate with malignant behaviors in breast cancer**

Xuehao Zhou, Yu Wang, Qingling Xu, Xiang Ao, Mengmeng Chen, Bingqiang Zhang, Ying Liu

Correspondence:

Bingqiang Zhang ([zhangbq@ruisidechina.com](mailto:zhangbq@ruisidechina.com)); Ying Liu (liuying_hero@163.com)


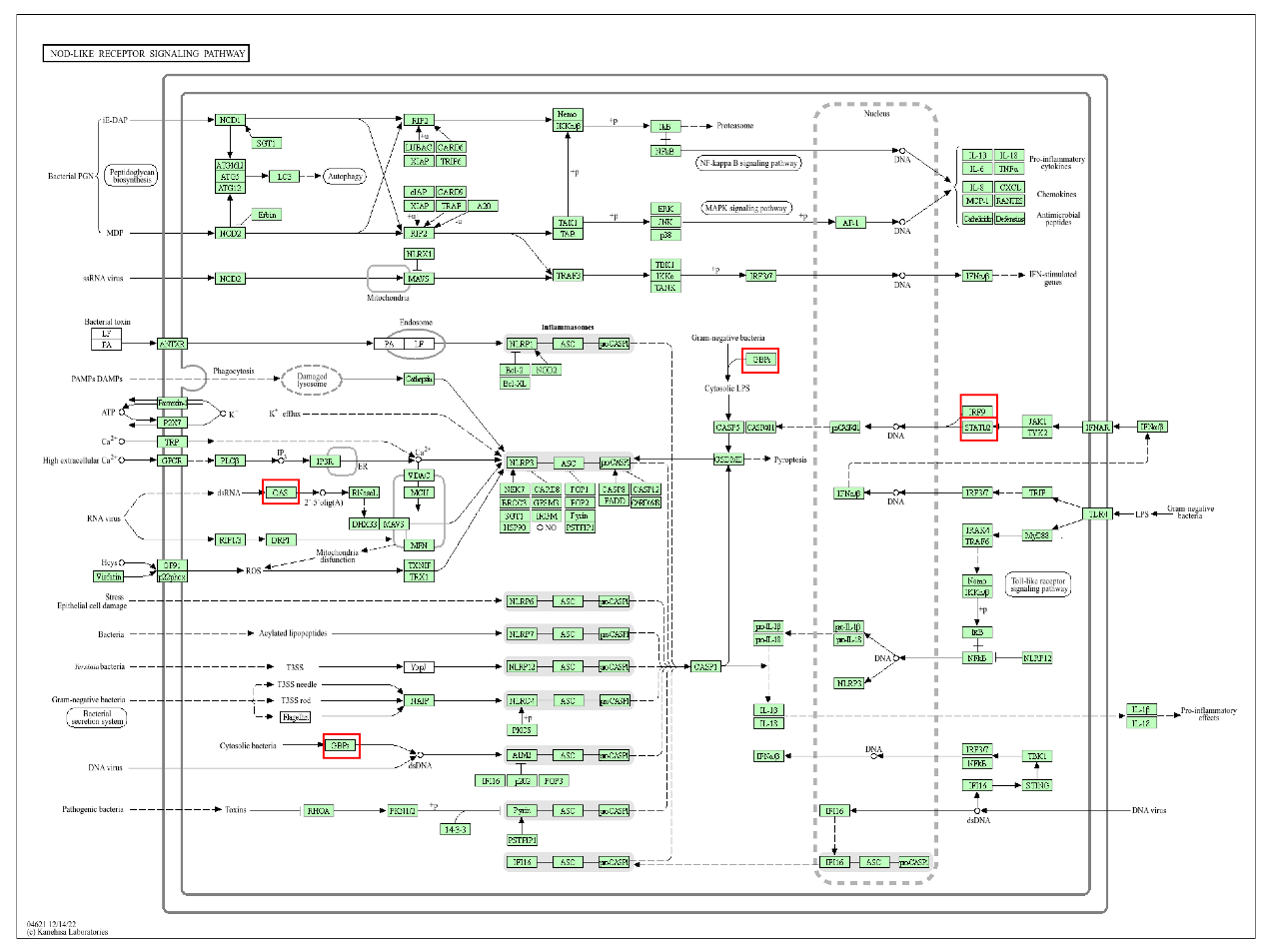


**Figure S1.** Visualization genes on NOD-like receptor signaling pathway map. NOD, nucleotide-binding and oligomerization domain.


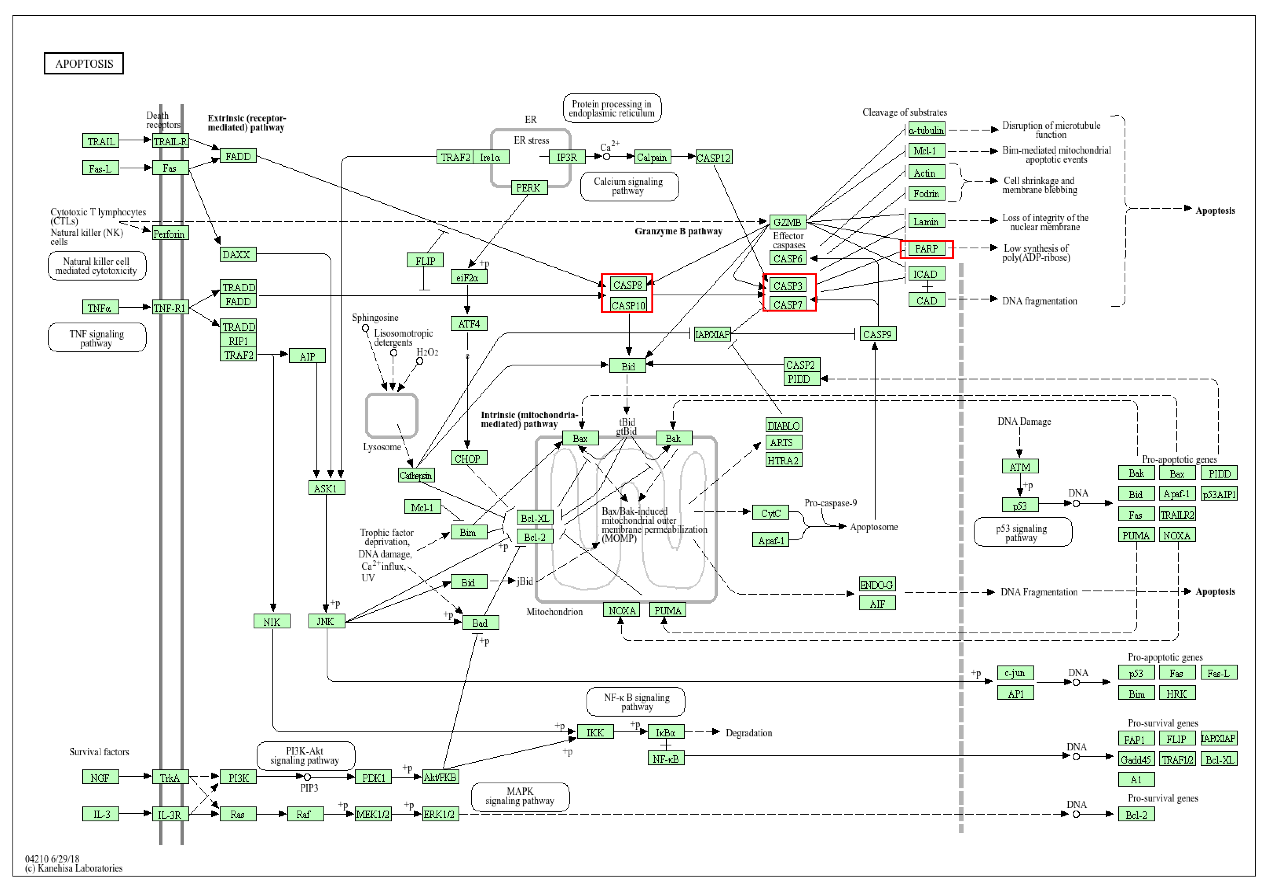


**Figure S2.** Visualization genes on apoptosis map.
