# Supplementary figures and images for: BALs are prognostic biomarkers and correlate with malignant behaviors in breast cancer
Source: BMC Cancer. 2025 Jul 24;25:1205. doi: 10.1186/s12885-025-14576-0 (PMC12288338; doi:10.1186/s12885-025-14576-0)

Figure 8B

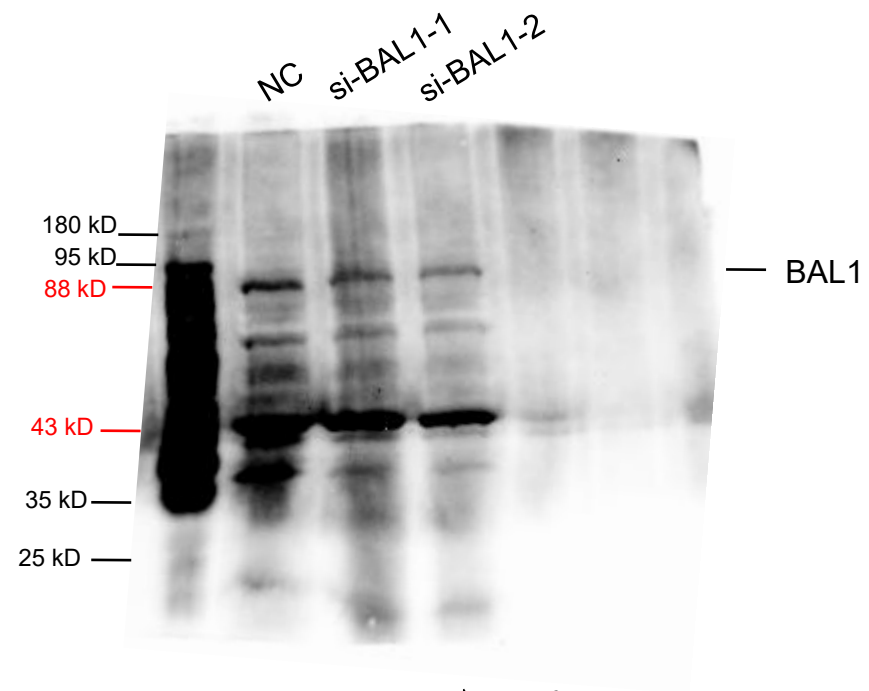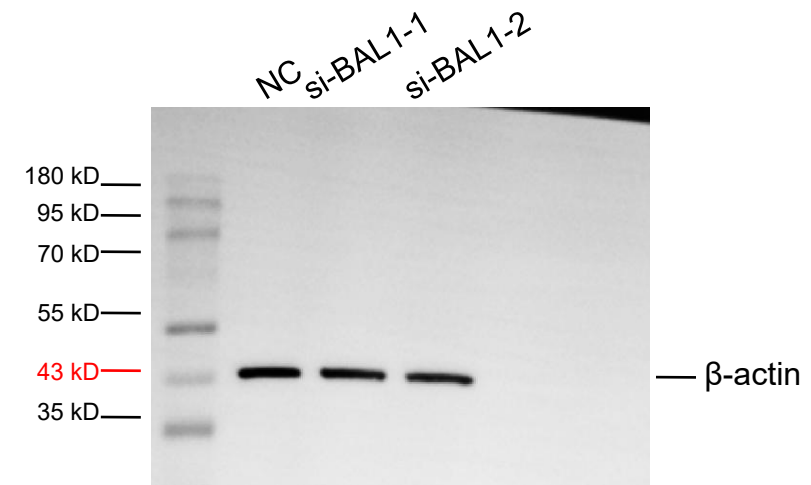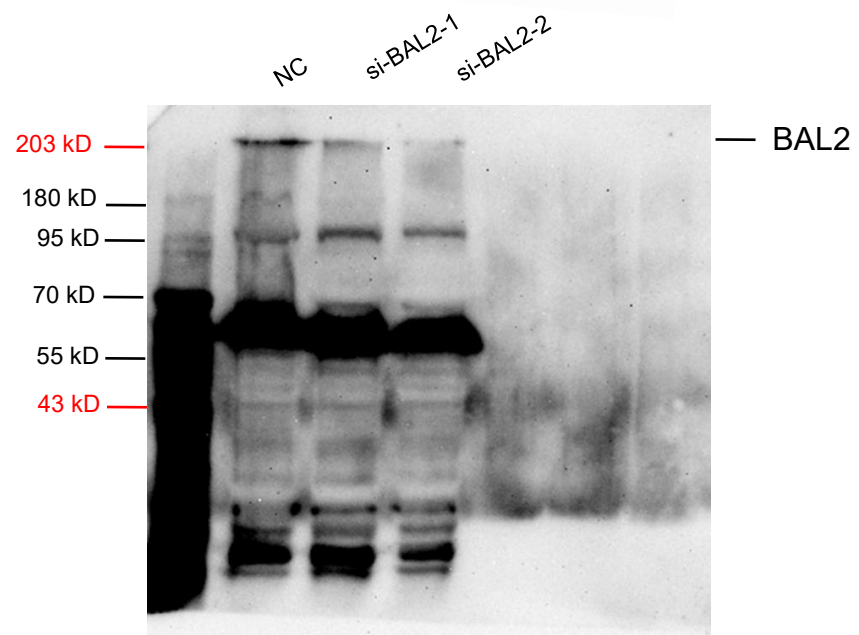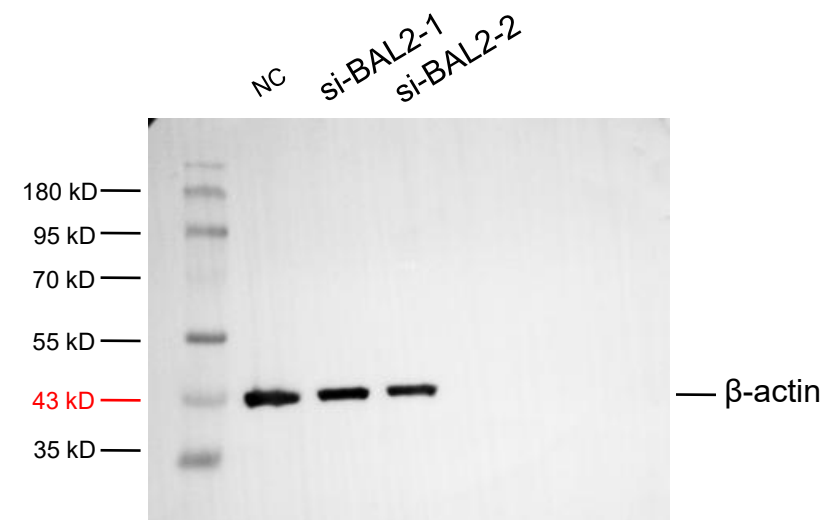

Figure 9A

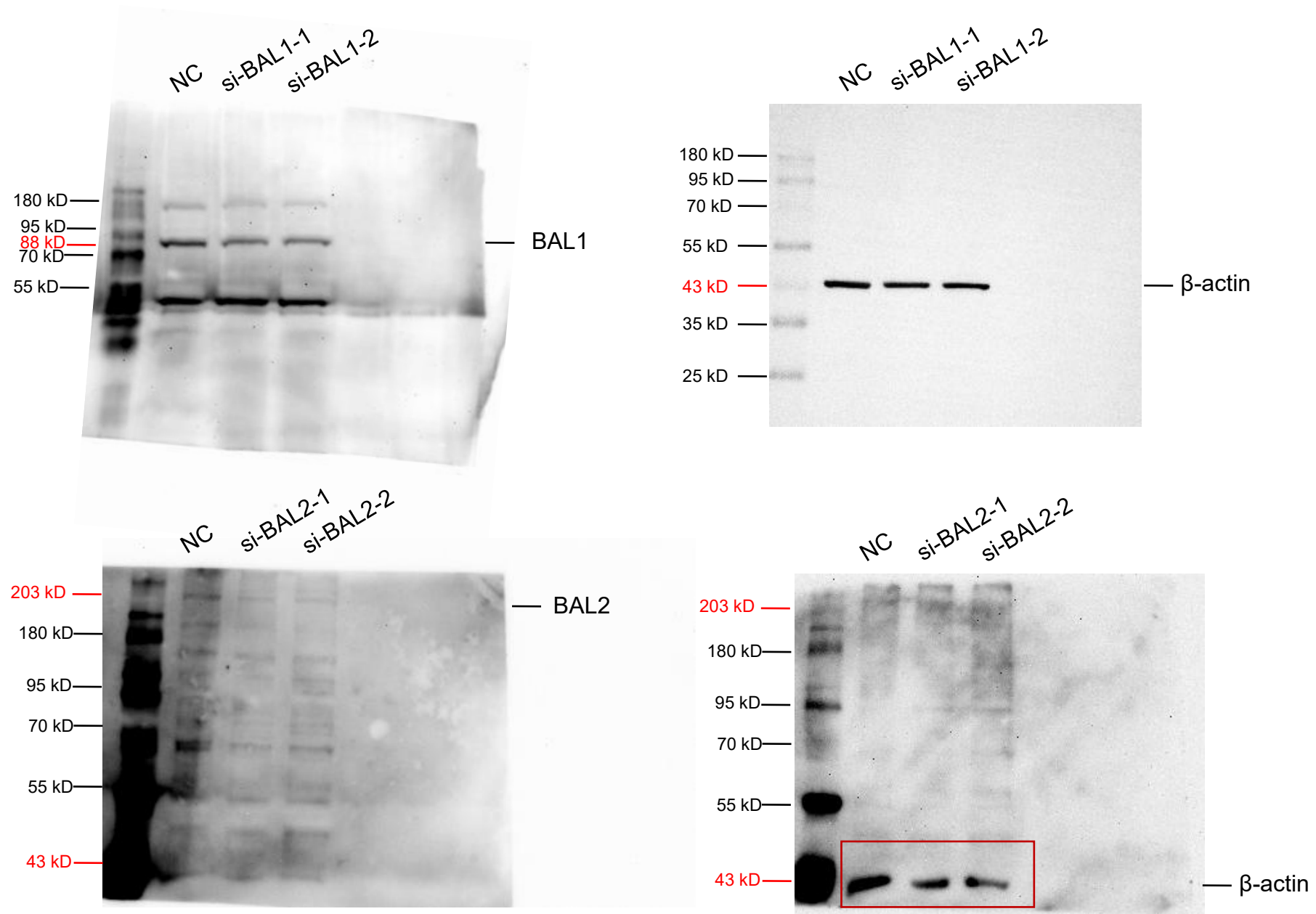

Supplement: Supplementary file 2 — Supplementary Material 2. [file 12885_2025_14576_MOESM2_ESM.pdf]
